# Micro- and mesozooplankton successions in an Antarctic coastal environment during a warm year

**DOI:** 10.1371/journal.pone.0232614

**Published:** 2020-05-14

**Authors:** Maximiliano D. Garcia, M. Sofia Dutto, Carlo J. Chazarreta, Anabela A. Berasategui, Irene R. Schloss, Mónica S. Hoffmeyer

**Affiliations:** 1 Instituto Argentino de Oceanografía (IADO-CONICET), Universidad Nacional del Sur (UNS), Bahía Blanca, Argentina; 2 Instituto Antártico Argentino (IAA), Buenos Aires, San Martín, Argentina; 3 Centro Austral de Investigaciones Científicas (CADIC-CONICET), Ushuaia, Argentina; 4 Universidad Nacional de Tierra del Fuego, Antártida e Islas del Atlántico Sur (UNTdF), Ushuaia, Argentina; 5 Facultad Regional Bahía Blanca, Universidad Tecnológica Nacional (UTN), Bahía Blanca, Argentina; Universidade de Aveiro, PORTUGAL

## Abstract

The rapid increase in atmospheric temperature detected in the last decades in the Western Antarctic Peninsula was accompanied by a strong glacier retreat and an increase in production of melting water, as well as changes in the sea-ice dynamic. The objective of this study was to analyze the succession of micro- and mesozooplankton during a warm annual cycle (December 2010-December 2011) in an Antarctic coastal environment (Potter Cove). The biomass of zooplankton body size classes was used to predict predator-prey size relationships (i.e., to test bottom-up/top-down control effects) using a Multiple Linear Regression Analysis. The micro- and mesozooplanktonic successions were graphically analyzed to detect the influence of environmental periods (defined by the degree of glacial melting, sea-ice freezing and sea-ice melting) on coupling/uncoupling planktonic biomass curves associated to possible predator-prey size relationship scenarios. At the beginning of the glacial melting, medium and large mesozooplankton (calanoid copepods, *Euphausia superba*, and *Salpa thompsoni*) exert a top-down control on Chl-*a* and microzooplankton. Stratification of the water column benefitted the availability of adequate food-size (Chl-*a* <20) for large microzooplankton (tintinnids) development observed during fall. High abundance of omnivores mesozooplankton (*Oithona similis* and furcilia of *E*. *superb*a) during sea-ice freezing periods would be due to the presence of available heterotrophic food under or within the sea ice. Finally, the increase in microzooplankton abundance in the middle of spring, when sea-ice melting starts, corresponded to small and medium dinoflagellates and ciliates species, which were possibly part of the biota of sea ice. If glacier retreat continues and the duration and thickness of the sea ice layer fluctuates as predicted by climate models, our results predict a future scenario regarding the zooplankton succession in Antarctic coastal environments.

## Introduction

In the Western Antarctic Peninsula (WAP) a rapid increase in atmospheric temperature has been detected in the last decades [1,2] accompanied by a strong warming of the upper ocean [3]. Some of the most notorious effects of this warming are glacier retreat and the increase in production of melting water from the glacial systems [4,5], as well as changes in the sea-ice dynamic [6–8].

The decadal temperature changes in the WAP are not only associated with the drivers of global temperature change but also reflect the extreme natural internal variability of the regional atmospheric circulation [9]. These regional changes are strongly affecting the physical and chemical properties of the water column [10]. For instance, glacier run-off transports high amounts of sedimentary material affecting light penetration and changing the optical conditions for phytoplankton photosynthesis [11]. Also, meltwater inflow favors water column stratification, especially in shallow coastal environments of the WAP [12], thus modifying the phytoplankton composition [13,14]. In addition, the strong trend toward an early disappearance of sea ice [6,15,16] could reduce the stratification of the water column and the magnitude of phytoplankton bloom the following spring [17]. These changes ultimately affect the habitat conditions for the micro- and mesozooplankton, hence their distribution and composition [18,19].

Global warming could additionally, change the phenology, or the time of occurrence within a succession of planktonic organisms, decoupling top-down control by microzooplankton on phytoplankton [20,21]. Microzooplankton plays a key role in the transfer of organic matter from pico- and nanoplankton to mesozooplankton [22,23]. Micro- and mesozooplankton responses to a warming scenario are often complex and vary according to the body size relations in the predator-prey interactions [24].

In Potter Cove (PC), at the South-West of King George Island (KGI/Isla 25 de Mayo), the addition of water from melting of the Fourcade Glacier has a significant impact on the temperature, salinity, and hence, stratification, and turbidity of the water column [11,12]. In this area, the temporal and spatial pattern of these physical variables, along with suspended particulate matter (SPM) and chlorophyll-*a* (Chl-*a*) concentrations have been studied from 1991 to 2009 by Schloss et al. [12] and Bers et al. [25] in relation to the local air temperature and winds, as well as zonal sea-ice cover. The last authors reported the existence of abrupt changes in sea surface temperature and salinity mostly related to climate cycles under the influence of the Southern Annular Mode (SAM) and El Niño Southern Oscillation (ENSO). These drivers influence micro and mesozooplankton assemblage dynamics both directly and indirectly [26–28] in PC during summer due to changes in the oceanographic conditions in the water column and the quality of the food available.

In this frame, here we present the first study of the succession of micro- and mesozooplankton in relation to glacier melting and sea-ice dynamics during an annual cycle in PC. The study was conducted in 2011, a year in which average air temperature (-2.36°C) was higher than the average of the previous 60 years (-2.5°C). We hypothesize that 1) variations in wind, glacial melting, and sea ice cover are the main drivers affecting the spatial and temporal dynamics of micro- and mesozooplankton in PC, determining differences in abundance, biomass, and taxonomic composition, and 2) top-down control exerted by zooplankton is linked to the predator-prey size relationships.

## Materials and methods

### Study area and sampling activities

This study was carried out at Potter Cove (PC, KGI, South Shetland Islands, Antarctica, 62.14°S, 58.38°W) by the Argentine Carlini Station (formerly Jubany) from December 2010 to December 2011. The cove is a fjord-like, shallow coastal environment of 2.5 km wide by 4 km long, with maximum depths > 100 m in the outer zone and < 50 m in the inner zone [11,29].

**Fig 1:**
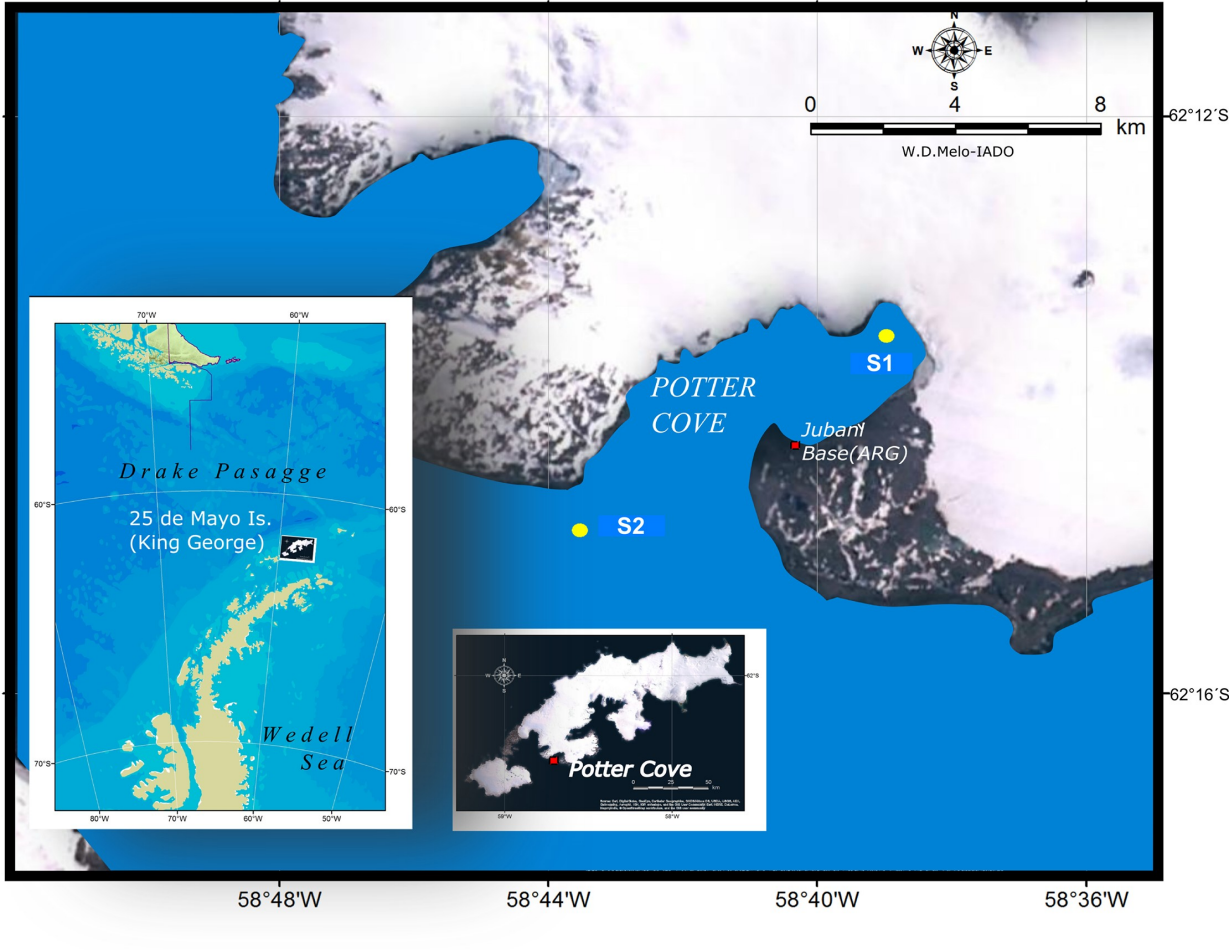
Study area (Potter Cove; Isla 25 de Mayo/King George Island). Location of the sampling sites: Site 1 (S1) was located at the inner zone close to the base of Fourcade Glacier, whereas Site 2 (S2) was located at the outer zone close to Maxwell Bay. Images are LANDSAT 8 OLI / TIRS, obtained from the US Geological Survey (https://earthexplorer.usgs.gov) and using ArcGis 10.1 software.

Sampling was carried out at two sites and two depths differently impacted by glacial melting [12]. Site 1 (S1) was located close to the base of Fourcade Glacier at the head of the inner cove, a zone consistently exposed to glacial meltwater inputs, whereas Site 2 (S2) was located at the mouth of the cove close to Maxwell Bay. Both zones are approximately 4 km apart (Fig 1). Samples were obtained at surface (named Surface; 5 m in S1 and 10 m in S2) and at depths below the summer pycnocline (Deep; 20 m in S1 and 30 m in S2). The field studies did not involve endangered or protected species.

Sampling was performed on a biweekly basis in summer and once a month during the rest of the year, under daylight conditions (between 9 am to 6 pm). The methodologies used for samplings varied according to the conditions of the cove (i.e. navigable or frozen sea). When navigable (December 2010 –May 2011 and November–December 2011), samples were collected from a Zodiac and when the cove was frozen (June–October 2011), sampling was performed from a hole in the sea-ice at S1. In these cases, sea ice instability conditions prevented the access to S2, except in July, despite the high degree of freezing of the inner zone. At each sampling event, density, salinity, temperature and turbidity profiles, from 30 m to the surface were obtained using a Seabird CTD (19 plus V2). The surface mixed layer depth (Zm) was estimated as the depth at which the gradient in density (σt) over a 1 m depth interval was >0.03 m^-3^ (threshold gradient method: [30,31]).

To analyze microzooplankton, SPM and Chl-*a*, 5 L water was collected with a Niskin bottle from surface and deep layers in the two sites. Microzooplankton samples for qualitative analyses were additionally obtained using 20 μm mesh net and short and slow horizontal tows. Mesozooplankton samples were collected by 2 knots-horizontal tows at the two depths using a 200 μm mesh net with a flow meter fixed to the mouth net. SMN (Meteorological National Service) office (named Estación Meteorológica Jubany) in the Argentine Carlini Station provided the meteorological data for the studied period. The extent of sea-ice in the cove was estimated by observational records (Garcia M.D., pers. observ.). The duration of sea-ice cover is defined as the difference between dates of sea-ice retreat and advance, corresponding to the last and first day of the year in which sea-ice concentration was higher than 15% for at least five consecutive days in the region [6]. The approximate thickness of the sea ice layer was also recorded through measurements of the depth of the holes made to perform the samplings.

### Laboratory activities

The SPM, organic (OM), and inorganic matter (IM) concentrations, were calculated following the gravimetric technique [32] with modifications accordingly [33]. For this, 0.5–1 L seawater was filtered through pre-combusted and pre-weighed GF/F filters. Filtered sediments were rinsed twice with distilled water to remove salts, dried for 24 h at 60°C, and weighed to obtain the weight of total SPM. Filters were then combusted for 5 h at 500°C and weighed again, thus obtaining the weight of the IM and by difference, the weight of the OM. Total Chl-*a* concentration was determined by filtering 0.5–1 L through GF/F filters. Additionally, 0.5 L samples were pre-filtered through a 20 μm pore diameter mesh to calculate the concentration of Chl-*a* corresponding to a fraction smaller than 20 μm (Chl-*a* <20) and filtered again through GF/F filters. The concentration of Chl-*a* greater than 20 μm (Chl-*a* >20) was calculated by difference with total Chl-*a*. Photosynthetic pigments were extracted during 24 h at 4°C under dark conditions with 90% acetone and read on a Shimadzu UV160A spectrophotometer. Chl-*a* concentration was estimated following Strickland and Parsons [34] after correction for phaeopigments.

Microzooplankton samples (250 ml) were preserved with acid Lugol’s solution (2% f.c.). For the estimation of microzooplankton abundance (ind L^-1^) 100 ml of a subsample was left for at least 24 h in a sedimentation chamber prior to the analysis following the Utermöhl method using an inverted microscope [35]. To estimate micozooplankton biomass, measurements of length and width were made on 30 individuals of each taxonomic group and these measurements were used to calculate cell volume (V; μm^3^) by approximation of each organism to a standard geometric configuration of similar characteristics [36,37]. Cell carbon concentration (μg C ind^-1^) was calculated using suitable conversion factors for each taxonomic group: aloricate ciliate (0.19 x V; [38]), tintinnids (0.053 + 444.5 x V; [39]), dinoflagellates (0.216 x V ^0.939^; [40]), rotifers (0.052 x V; [41]), and nauplii of copepods (0.08 x V; [42]). The estimated value of individual biomass was multiplied by its abundance to obtain the biomass of each group expressed in values of μg C L^-1^.

Mesozooplankton samples were preserved in 4% neutralized formaline (f.c.) and then analyzed under stereo microscopes by aliquots (10% of the sample) or total counting to obtain mesozooplankton abundance (ind m^-3^). The main taxonomic groups Copepods and Euphausiids were identified at a species level; while the rest of the main taxonomic groups (those that represented at each sampling site less than 2% of the total mesozooplankton abundance: Hydromedusae, Ctenophora, Siphonophorae, Amphipoda, Chaetognatha, Ostracoda, Pteropoda, and Salpida) were grouped as "others taxa" and only Pteropoda and Salpida were identified at species level. To estimate mesozooplankton biomass, the individual dry/wet weight of the most representative taxa (those that represented more than 1% of the total mesozooplankton biomass) were either taken from the literature (e.g. [43–48]) or calculated by applying body size–carbon content relationship equations (e.g. [49–51]). Carbon values (μg C L^-1^) in turn, were derived applying conversion factors from the literature [52,53].

### Data analyses

To analyze meteorological and oceanographic data variability among seasons, sampling sites (S1 and S2), and depths (Surface and Deep) non-parametric Kruskal–Wallis tests were performed on seasonal averages of wind, air and water temperature, salinity, turbidity, Chl-*a*, and SPM, owing to the rejection of normality and homoscedasticity assumptions. In order to observe any pattern of spatial and temporal distribution of zooplankton biomass and composition between sites, depth, seasons, and environmental periods (defined by the degree of glacial melting, sea-ice freezing and sea-ice melting), we first analyze the data using a Cluster ordination analysis, with a One-way analysis of similarity (ANOSIM) to determine the significance of spatial and temporal dynamic of zooplankton, and a similarity percentage analysis (SIMPER) to observe the percentage contribution of each specie to either the similarities within a given group or dissimilarities between groups. These multivariate analyses were performed using PRIMER V6 software. After that, multivariate redundancy analyses (RDAs) were performed to explore the temporal associations between the biomass of micro-and mesozooplankton taxonomic groups along gradients of Chl-*a* and environmental data in the four spatial scenarios over the annual cycle studied: S1-Surface, S1-Deep, S2-Surface, and S2-Deep. These statistical analyses were performed after confirming through a preliminary detrended correspondence analysis (DCA) that the length of the gradient in units of standard deviation obtained was lower than 4 [54]. RDA and significance of the first two axes were tested by a Monte-Carlo permutation test using CANOCO Version 4.5 software.

To detect local trends, the biotic and abiotic dataset obtained in PC during the sampling period was analyzed. The biomass of plankton body size classes (determined by zooplankton size structure) was used to predict predator-prey size relationships (i.e., to test bottom-up/top-down control effects) using a Multiple Linear Regression Analysis (MLRA). Microzooplankton was classified into volume-size classes (μm^3^ cell^-1^): <10^3^, 10^3^−10^4^, 10^4^−10^5^, and > 10^5^, while mesozooplankton were classified into length-size classes (μm): 10^3^, 10^3^−10^4^, 10^4^−10^5^, and > 10^5^. Phytoplankton biomass (as Chl-*a* concentration) was classified as Chl-*a* <20 and Chl-*a* >20. Micro- and mesozooplankton size-classes were the dependent variables, whereas Chl-*a*, and micro- and mesozooplankton size-classes (i.e., those that could have a role of prey or predator) acted as explanatory variables. These statistical analyses were performed using STATISTICA version 7. The micro- and mesozooplanktonic successions in terms of size structure and total biomass were graphically analyzed, to detect the influence of environmental periods (glacial melting, glacial melting-stratification, fall, sea-ice freezing and sea-ice melting) on the plankton assemblages and to identify trophic relationships scenarios (coupling/uncoupling) in the planktonic biomass curves.

## Results

### Dynamic of meteorological and oceanographic conditions

The average air temperature in 2011 (−2.36 ±5.30°C) varied from −10.02°C (±5.81) in July to 2.93°C (±1.23) in February. Maximal average wind speed was recorded in spring (34.74 ±14.71 Km h^-1^) and fall (34.02 ±13.13 Km h^-1^). October was the windiest month (40.38 ±16.83 Km h^-1^). West winds were dominant during the study period while the maximum wind speeds were from NW–W. Observations and photographic records obtained at PC indicated that the sea-ice cover lasted 143 days, from day 158 (beginning of June) to day 301 (end of October). The maximum thickness of the sea ice layer was observed in August with 1.5 m in the inner zone and 0.50 m in the outer zone. During the end of August and the beginning of September the entire cove was frozen. In mid-October, the sea-ice cover began to decline, in thickness and extent, only the inner zone remained frozen.

The average sea water temperature was significantly lower at S1 (0.07 ±1.33°C) than at S2 (0.76 ±1.07°C; p <0.05). Salinity showed a similar, but not significantly, pattern with lower average value at S1 (33.71 ±1.12) than at S2 (34.00 ±0.20). Turbidity at S1 was significantly higher than at S2 (5.13 ±7.11 NTU and 0.83 ±0.89 NTU, respectively; p <0.05). At S1 significant differences between the two layers of the water column were found showing higher values of turbidity in the surface (7.85 ±8.06 NTU) than in the deep layer (2.41 ±4.86 NTU; p <0.05) and in opposite trend, higher values of salinity were recorded in the deep (34.05 ±0.09) than in the surface layer (33.38 ±1.53; p <0.05). Several variables showed clear seasonal patterns. Significantly higher average values of water temperature, turbidity, OM, and Chl-*a* were recorded in summer (1.40 ±0.35°C, 5.22 ±7.31 NTU, 2.31 ±1.38 mg L^-1^, and 1.37 ±1.02 μg L^-1^, respectively) than in winter (-1.56 ±0.10° C, 0.65 ±0.69 NTU, 0.93 ±0.52 mg L^-1^, and 0.09 ±0.02 μg L^-1^, respectively; p <0.05). In contrast, salinity showed the highest average value in winter (34.30 ±0.40; p <0.05) compared with the remaining seasons.

**Fig 2:**
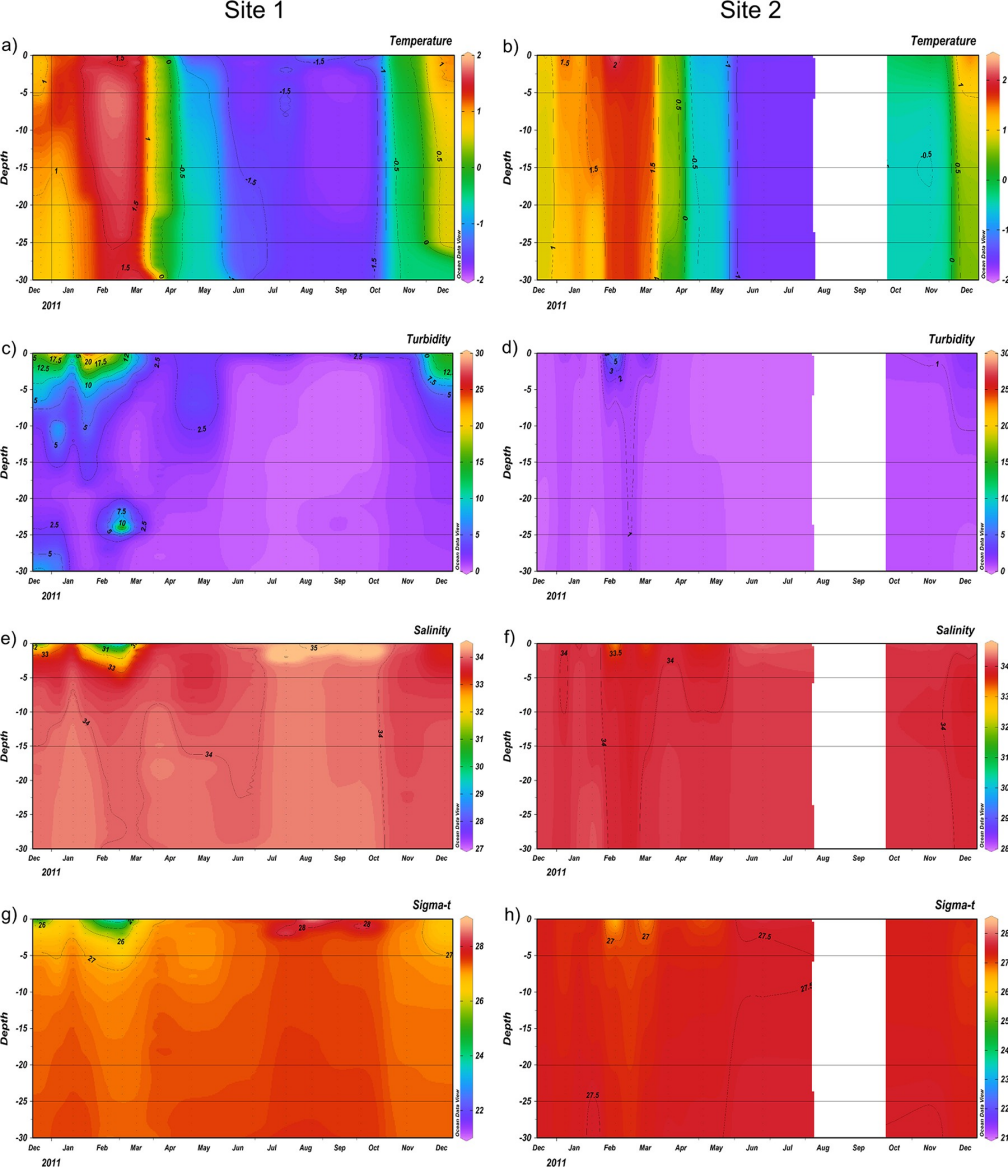
The CTD profiles. Spatial and temporal dynamics of oceanographic variables at Site 1-Site 2; showing water temperature (Fig 2A and 2B), turbidity (Fig 2C and 2D), salinity (Fig 2E and 2F), and sigma (Fig 2G and 2H).

The CTD profiles (Fig 2A–2H) showed the spatio-temporal dynamics of the oceanographic variables. At S1 during the summer, high vertical variability of water temperature (Fig 2A), turbidity (Fig 2C), salinity (Fig 2E), and sigma (Fig 2G) were observed. This hydrographic structure clearly indicated an intense glacial melting period and stratification of the water column at the end of summer (February and March) at S1.

### Zooplankton composition and environmental and trophic associations

**Fig 3:**
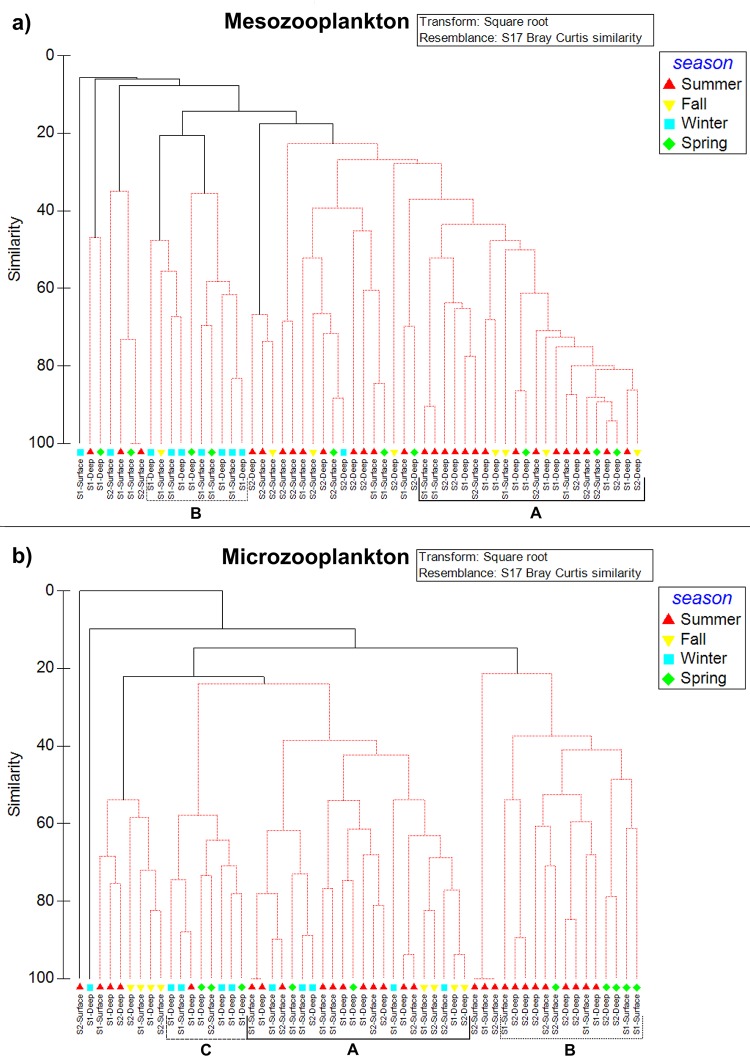
Cluster ordination analysis. Spatial and temporal dynamic of zooplankton biomass data sets of sampling sites and depths during a year in Potter Cove. Letters A, B, and C indicate the groups of samples in relation to their mesozooplankton (a), and microzooplankton (b) composition.

The Cluster ordination analysis generated two ordering graphs in which groups of samples related in terms of similarity in biomass and composition of microzooplankton and mesozooplankton (Fig 3). Mesozooplankton samples were grouped in: Group A (formed by samples from summer and S1-Deep) with 40 similarity, and Group B (consisting mostly of samples from winter and S1-Surface) which showed 20 similarity value (Fig 3A). Microzooplankton was grouped in: Group A (composed of superficial samples of Site 1 and Site 2), Group B (characterized by samples from winter and S1-Deep), both groups with 40 similarity values; and Group C (consisting mostly of samples from summer and spring and S2-Deep) showing 60 similarity value (Fig 3B). Results from the ANOSIM global test indicated that there were no significant differences in the composition of mesozooplankton and microzooplankton between sampling sites, between depths, and between spatial scenarios (Table 1). In contrast, temporal distribution was significantly different among environmental periods for microzooplankton and among seasons and environmental periods for mesozooplankton (Table 1). SIMPER analysis revealed that *Calanus propinquus* and *Euphausia frigida* (furcilia larvae) were the most discriminating taxa with a contribution higher than 24% and 15%, respectively to the dissimilarity of mesozooplankton temporal dynamics. *C*. *propinquus* and furcilia larvae of *E*. *frigida* contributed in 48.78% and 17.14%, respectively to the similarity within winter samplings; and in 40.89% and 29.72%, respectively to the similarity within Sea-ice freezing samplings. SIMPER also showed that the significant differences among environmental periods for microzooplankton were due to the presence of *Codonellopsis balechi*, the most discriminating taxon with a contribution always higher than 22% to the dissimilarity. *C*. *balechi* showed a contribution of 76.10% and 44.57% to the average of similarity of Glacial melting-stratification and Sea-ice freezing periods, respectively. In addition, others taxa which characterized environmental periods were: *Protoperidinum* aff. *concavum* (34.67% to the average of similarity of Fall samples), *Gyrodinium lachryma* (36.16% of Glacial melting), and *Strombidium* spp. (33.92% of Sea-ice melting).

**Table 1:** Results of one-way ANOSIM.

ANOSIM Factors	Microzooplankton		Mesozooplankton	
	Global R	Significance level *p*	Global R	Significance level *p*
Sites (S1—S2)	0.03	ns	0.04	ns
Depths (Surface—Deep)	0.001	ns	0.03	ns
Spatial scenarios (S1-Surface, S1-Deep, S2-Surface, S2-Deep)	0.03	ns	0.04	ns
Seasons (Summer, Fall, Winter, Spring)	0.08	ns	0.2	**0.01**
Winter VS Summer		ns		**0.001**
Winter VS Spring		ns		**0.01**
Environmental periods	0.3	**0.001**	0.1	**0.003**
Glacial melting VS Glacial melting-stratification		**0.001**		ns
Glacial melting VS Fall		**0.001**		ns
Glacial melting VS Sea-ice freezing		**0.001**		**0.001**
Glacial melting-stratification VS Sea-ice melting		**0.003**		ns
Fall VS Sea-ice freezing		**0.001**		**0.04**
Fall VS Sea-ice melting		**0.001**		ns
Sea-ice freezing VS Sea-ice melting		**0.003**		**0.001**
Glacial melting-stratification VS Sea-ice melting		ns		**0.002**

Showing the values corresponding to results derived from global tests (R and Significance level p) and the significant results (*p* < 0.05) derived from pairwise tests.

**Fig 4:**
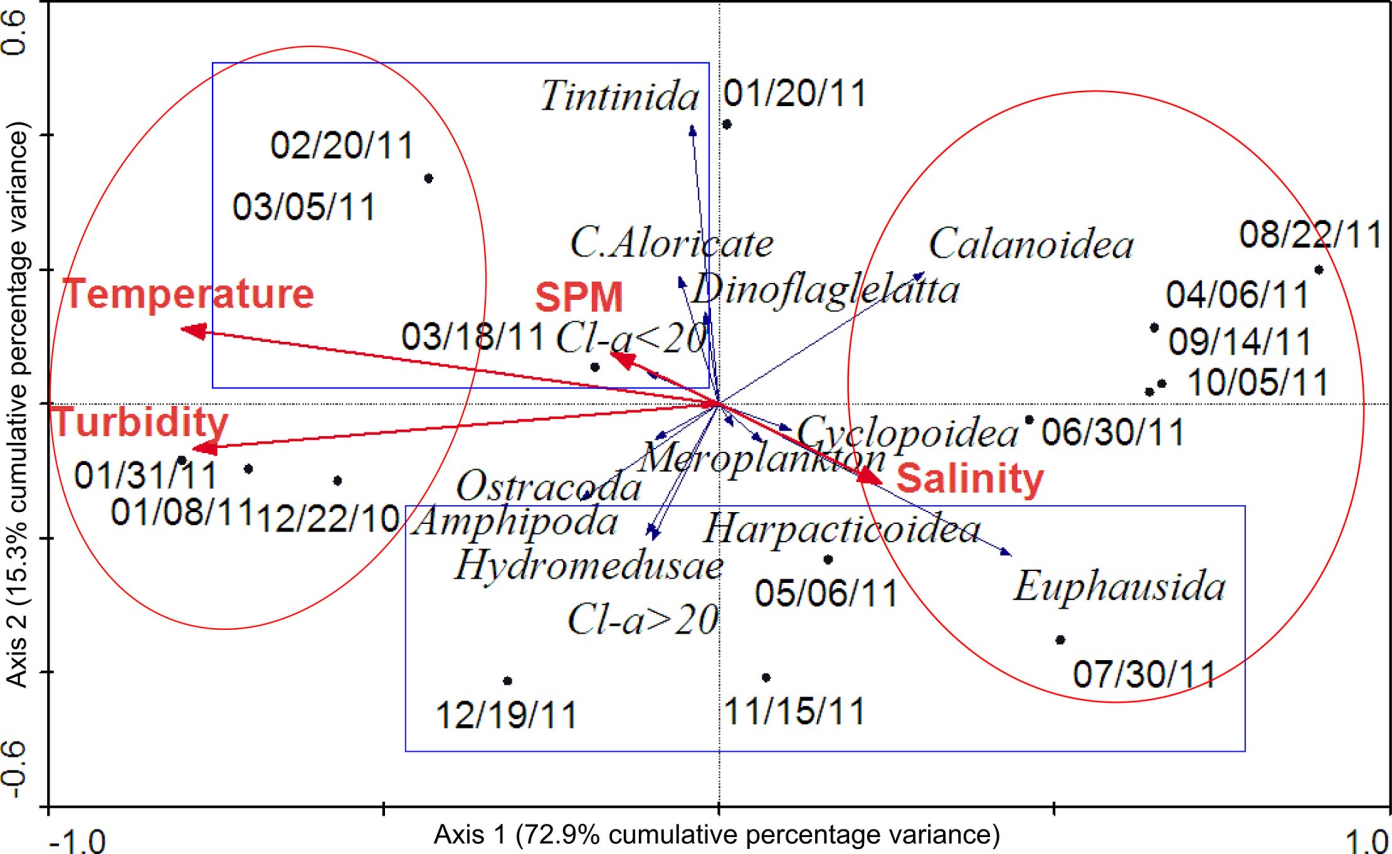
Redundancy analyses (RDA) results for Site 1-Surface of Potter Cove. The axis 1 shows a temporal environmental gradient with two distinguishable groups (red ellipsis) and the second axis shows two possible trophic associations (blue rectangles).

Considering the RDAs results, the only spatial scenario which showed statistical significance (Monte Carlo test p <0.05) was S1-Surface (Fig 4). In this case, the first two ordination axes, according to the species-environment variables’ relations, represented more than 88% of total variance. The variance inflation factor was less than 20 for the variables included in the RDAs (turbidity = 15.28, temperature = 6.94, salinity = 3.36, and SPM = 4.64). The species ordination on axis 1 was positively correlated to salinity (0.18) and negatively correlated to temperature (-0.61), turbidity (-0.60), and SPM (-0.12). The analysis of this axis indicated a temporal environmental gradient with two distinguishable groups (Fig 4, red ellipsis): the first one showed summer’s sampling dates associated with high values of turbidity and water temperature and low salinity. The second group was composed mainly by several sampling dates of winter, and high biomass values of calanoids and euphausiids, correlated to high salinity. The biological ordination on the second axis was positively correlated to temperature (0.10) and SPM (0.07) and negatively correlated to salinity (-0.11) and turbidity (-0.06). This axis showed two possible trophic associations (Fig 4, blue rectangles): a strong association between the biomass of microzooplankters and Chl-*a* <20 related to some summer sampling dates; and another group formed by different mesozooplankton taxa and Chl-*a* >20 associated to winter, fall, and spring sampling dates. In the other three spatial scenarios analyzed, RDAs were not statistically significant and only a temporal gradient was detected in S1-Deep where fall, winter and spring sampling dates were associated with high values of salinity and biomass of euphausiids.

### Predator–prey size relationship: Looking for local trends in the zooplankton succession

**Fig 5:**
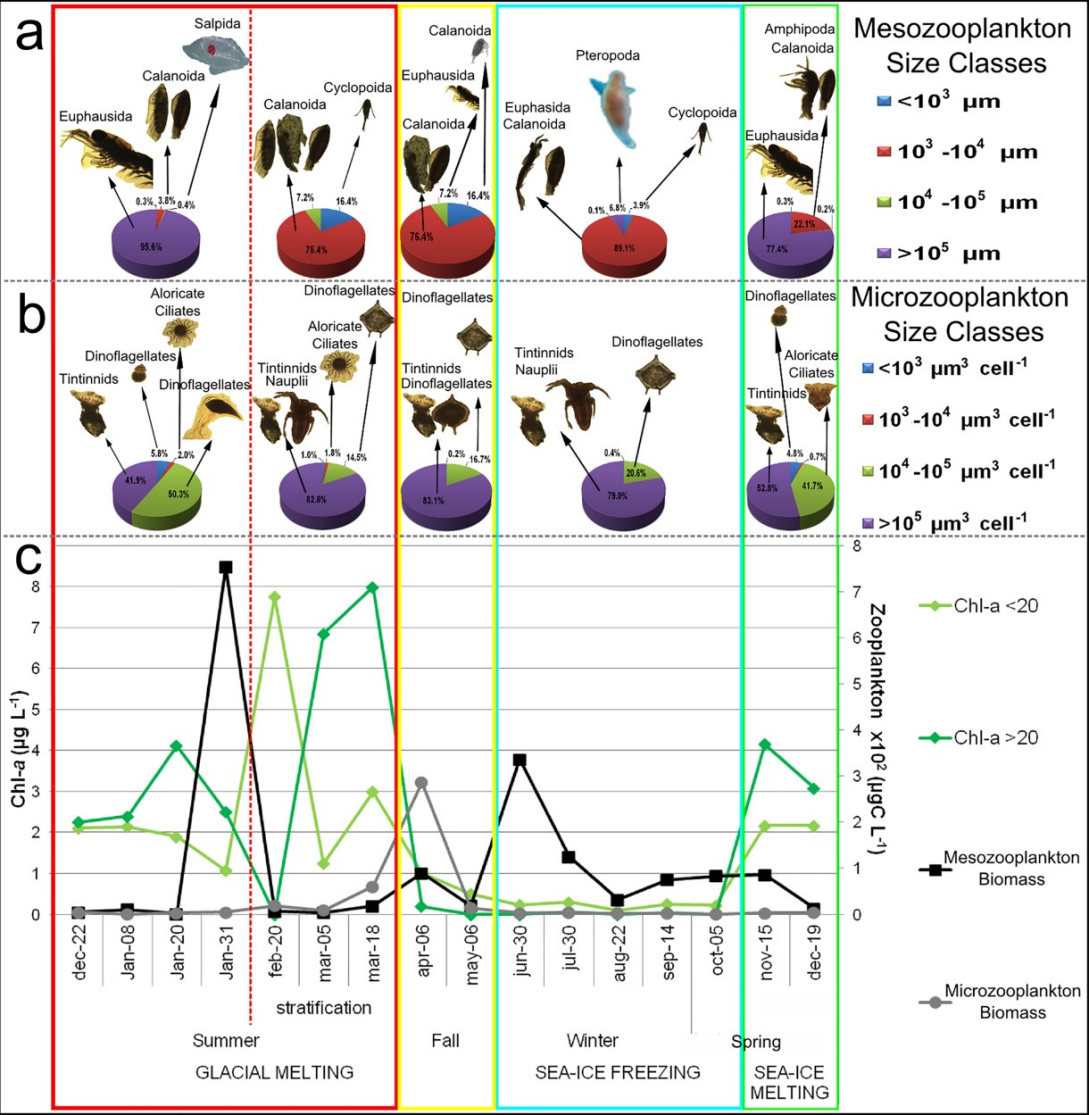
Zooplanktonic succession. Composition of mesozooplankton size classes (a) and microzooplankton size classes (b) during environmental periods. Temporal dynamics of micro- and mesozooplanktonic biomass and chlorophyll-*a* curves (c) identify trophic relationships scenarios (coupling/uncoupling) in an annual cycle on the plankton assemblages. The data presented in this figure correspond to the average of both stations and depths. For a better specific identification of the taxonomic groups and the most representative taxa see Table 2.

MLRA performed from zooplankton size-class structure (Table 2) resulted in 7 statistically significant models (4 for microzooplankton and 3 for mesozooplankton; Table 3). By relating the results of the MRLA with the coupling/uncoupling of the planktonic biomass curves (Fig 5), possible predator-prey size relationship scenarios were identified during five environmental periods. At the beginning of the period of glacial melting (December 2010 –January 2011), the maximum values of mesozooplankton biomass were identified with species of medium and large size (*E*. *superba* and *Salpa thompsoni)* followed by calanoid copepods. In these sampling dates, low values of Chl-*a* and microzooplankton biomass were observed graphically as an uncoupling scenario in relation to mesozooplankton biomass curves (Fig 5A, 5B and 5C). Negative relationships between mesozooplankton (10^4^−10^5^ and > 10^5^ μm) and possible prey (Chl-*a* <20, Chl-*a* >20, and microzooplankton >10^5^) were also identified (Table 3).

**Table 2:** Zooplankton size structure. Showing the percentage of representation of each size class in the total biomass and of each taxonomic group in each size class.

**Mesozooplankton Length Size Classes**	**Percentage of representation in Total Mesozooplankton Biomass**	**Taxonomic Groups**	**Percentage of representation in each size class**	**Most representative taxa**
**<10**^**3**^ **μm**	2.58	Cyclopoida	31.16	*Oithona similis*
		Calanoida	39.20	*Ctenocalanus citer*
		Harpacticoida	22.39	Ectinosomatidae
		Meroplankton	6.82	Veliger larvae (Gasteropoda)
**10**^**3**^**−10**^**4**^ **μm**	44.51	Calanoida	74.69	*Calanus propinquus*; *Calanoides acutus*; *Rhincalanus gigas*
		Euphausida	20.33	*Euphausia frigida* (Furcilia)
		Others taxa	4.90	Amphipoda
**10**^**4**^**−10**^**5**^ **μm**	1.25	Euphausida	69.96	*Euphausia superba* (juvenil)
		Others taxa	30.04	*Salpa thompsoni*; Chaetognatha
**>10**^**5**^ **μm**	51.66	Euphausida	94.85	*Euphausia superba* (adult)
		Others taxa	5.15	*Clione limacina*
**Microzooplankton Volume Size Classes**	**Percentage of representation in Total Microzooplankton Biomass**	**Taxonomic Groups**	**Percentage of representation in each size class**	**Most representative taxa**
**<10**^**3**^ **μm**^**3**^ **cell**^**-1**^	0.59	Dinoflagellates	100.00	*Gimnodinium* sp. 1; *Gyrodinium* sp.
**10**^**3**^**−10**^**4**^ **μm**^**3**^ **cell**^**-1**^	0.45	Dinoflagellates	32.68	*Mesodinium* sp.; *Katodinium* sp.
		Aloricate Ciliates	65.73	*Strombidium* aff. *epidemum*
**10**^**4**^**−10**^**5**^ **μm**^**3**^ **cell**^**-1**^	17.85	Dinoflagellates	75.24	*Protoperidinium* aff. *concavum*; *Preperidinium meunierii*; *Gyrodinium lachryma*
		Aloricate Ciliates	23.69	*Strombidium* spp.
		Tintinnids	1.07	*Codonellopsis glacialis*
**>10**^**5**^ **μm**^**3**^ **cell**^**-1**^	81.11	Dinoflagellates	29.38	*Protoperidinium* aff. *antarcticum*;
		Aloricate Ciliates	2.94	*Leegaardiella aff*. *elbraechteri*
		Tintinnids	59.35	*Codonellopsis balechi*
		Nauplii	8.10	Nauplii larvae (Copepoda)

**Table 3:** Multiple linear regression analysis (MLRA) models for zooplankton size-classes as dependent variables: Microzooplankton volume-size classes (MiVSC (μm^3^ cell^-1^): <10^3^, 10^3^−10^4^, 10^4^−10^5^, and > 10^5^) and Mesozooplankton length-size classes (MeLSC (μm): <10^3^, 10^3^−10^4^, 10^4^−10^5^, and > 10^5^) for the studied period (December 2010 –December 2011) in Potter Cove.

Dependent Variable		Models	R^2^	p
**MiVSC (μm**^**3**^ **cell**^**-1**^**)**	**<10**^**3**^ **=**	0.1 Chl-*a* <20 + 0.01 MiVSC 10^4^−10^5^ + 0.01 MeLSC>10^5^–0.1 MiVSC 10^3^−10^4^ + 0.01 MeLSC <10^3^–0.01 MeLSC 10^3^−10^4^	**0.61**	**
	**10**^**3**^**−10**^**4**^ **=**	0.21 Chl-*a* <20–0.69 MiVSC<10^3^–0.02 Chl-*a* >20 + 0.01 MeLSC>10^5^ + 0.01 MiVSC 10^4^−10^5^	**00.51**	**
	**10**^**4**^**−10**^**5**^ **=**	0.12 MiVSC>10^5^	**0.55**	**
	**>10**^**5**^ **=**	4.39 MiVSC 10^4^−10^5^–0.24 MeLSC 10^3^−10^4^	**0.48**	**
**MeLSC (μm)**	**<10**^**3**^ **=**	0.06 MeLSC 10^3^−10^4^ + 2.60 MiVSC<10^3^ + 0.20 Chl-*a* >20	**0.90**	**
	**10**^**3**^**−10**^**4**^ **=**	114.06 MeLSC<10^3^–0.26 MiVSC>10^5^–4.25 Chl-*a* >20–5.34 Chl-a<20 + 1.03 MiVSC 10^3^−10^4^	**0.92**	**
	**10**^**4**^**−10**^**5**^ **=**	- - 0.34 Chl-*a* >20	**0.01**	**ns**
	**>10**^**5**^ **=**	1929.54 MeLSC<10^3^–104.59 Chl-*a* <20–1.78 MiVSC>10^5^ + 118.30 MiVSC 10^3^−10^4^	**0.51**	**

The percentage of explanation of the variance is shown taking into account the number of variables of the model (adjusted R^2^) and the critical values for statistical significance were p <0.05 (*) and p <0.01 (**).

The increase of the smallest microzooplankters (<10^3^ and 10^3^−10^4^ μm^3^ cell^-1^, observed only in the first period of glacial melting) composed of athecate dinoflagellates (*Gimnodinium* sp. and *Gyrodinium* sp.) and aloricate ciliates (*Mesodinium* sp.; Table 2) together with Chl-*a* <20 were graphically observed during the beginning of stratification periods of the water column in summer (February-March; Fig 5B and 5C). These microzooplankton size classes showed a positive relationship with Chl-*a* <20 (Table 3) and a coupling (Fig 5B and 5C). In this period, the start of the increase in mesozooplankters <10^3^ μm (since feb-20) and 10^3^−10^4^ μm (in March) was mostly represented by copepods (such as: *O*. *similis*, *C*. *propinquus*, *Calanoides acutus*, and *Rhincalanus gigas*; Table 2) (Fig 5A). The smallest class of mesozooplankton showed positive relations with <10^3^ and 10^3^−10^4^ μm^3^ cell^-1^ classes of microzooplankton and Chl-*a* >20. Also, a negative relationship between mesozooplankton 10^3^−10^4^ μm and microzooplankton <10^3^ μm^3^ cell^-1^ (Table 3) was visible in March (Fig 5A 5B and 5C). However; the dominance of small mesozooplankton groups kept low biomass values in this period. At the end of the water stratification (end of March), the increase of microzooplankton biomass was represented exclusively by species of 10^4^−10^5^ and >10^5^ μm^3^ cell^-1^ (*e*.*g*. *Protoperidinium* and *C*. *balechi*; Table 2).

During fall, the highest value of microzooplankton biomass (April) accompanied by an increase of large copepods of 10^3^−10^4^ μm (coupling) and a decrease of Chl-*a* was observed (uncoupling; Fig 5A and 5C). A negative relationship between mesozooplankters of 10^3^−10^4^ μm and Chl-*a* <20, Chl-*a* >20 and microzooplankton >10^5^ μm^3^ cell^-1^ (Table 3) was recorded.

During the whole period of sea-ice freezing, decreases of phytoplankton and microzooplankton biomass were observed (Fig 5C). Of these, phytoplankton obtained higher values of the fraction smaller than 20 microns, while the micro-heterotrophs were mainly represented by >10^5^ and 10^4^−10^5^ μm cell^-1^ such as nauplius larvae of copepods, *C*. *balechi*, and species of the genus *Protoperidinium* (Fig 5B, Table 2). On the contrary, mesozooplankton biomass was high and showed the second highest values of the annual cycle at the beginning of this period (Jun-30; Fig 5C). The sea-ice freezing period was dominated by mesozooplankton of 10^3^−10^4^ μm (mostly *C*. *propinquus* and furcila larvae of euphausiids) with <10^3^ μm (*O*. *similis*) and >10^5^ μm classes (*Clione limacina*) as accompanying groups at the beginning and end of this period, respectively (Fig 5A, Table 2). According to MRLA, mesozooplankton 10^3^−10^4^ μm presented a negative relationship with Chl-*a* <20, Chl-*a* >20 and microzooplankters >10^5^ μm cell^-1^ (Table 3).

In the middle of spring, when sea-ice melting starts, a considerable increase of Chl-*a* was observed; mesozooplankton biomass showed a slight increase and a subsequent decrease in November and December, respectively (Fig 5C). Mesozooplankton biomass was first represented by large- sized groups (>10^5^ μm) as adults of *E*. *superba* and amphipods and then succeeded by 10^3^−10^4^ μm (hydromedusae and calanoid copepods) and 10^4^−10^5^ μm classes (salps, Fig 5A, Table 2). Microzooplankton biomass increased slightly during this period as a result of the increase in the abundance of small dinoflagellates such as *Gymnodinium* sp. and *Gyrodinium* sp., and intermediate-sized ciliates such as *Strombidium* spp. and *Leegardiella* spp. (Fig 5B). Various mesozooplankton size classes (10^3^−10^4^, 10^4^−10^5^, and >10^5^ μm) presented negative relationships with large phytoplankton (Chl-*a* >20) and large microzooplankton (>10^5^ μm cell^-1^; Table 3).

## Discussion

### 2011, a warm year

In 2011, the variation in air temperature in Potter Cove was extreme, with both a warmer summer and a colder winter as compared to climatic values for the previous two decades [12]. Furthermore, the mean air temperature in the studied period (-2.36°C) was slightly higher than the value recorded by Kejna et al. [55] for KGI between the years 1948–2011 (-2.5°C). Western winds, dominated the pattern, as in the previous years [12]. In the WAP strong westerly winds are related to climatic variability such as the prevalence positive conditions of SAM [6,12]. According to [2], the change in dominance of winds direction from east to west during 2011 would be related to the end of the great La Niña event in the middle of that year.

In our study, periods of high contribution of glacial meltwater were associated to a decrease in salinity, increase in turbidity (correlated with SPM), and stratification of the water column. Similar oceanographic conditions were observed in PC during summer in superficial waters close to the glacier [12,25,56]. The discharge of glacial meltwater is accumulated into an extremely thin layer at the surface; this layer responds rapidly to changes in atmospherically-driven circulation to generate a strongly pulsed outflow from the cove to the broader ocean [10]. But, according to Klöser et al. [29], the westerly winds favor the entry of water from outside the cove and water accumulates in the inner cove. Therefore, in PC, when prevailing winds were from the west sector, the water column stratification were favored [29,57].

The period in which PC remained covered by sea-ice during 2011 (143 days) was lower than the average recorded by Schloss et al. [12] but was still within the range (close to the minimum, 142 days in 2007) for the period 1991–2009. This low ice cover in 2011 was probably influenced by the strong western winds from mid-October that would have helped with the breakdown of sea ice from the outer sector of the cove. The large decrease in sea ice extent in September/October can be associated to a more positive SAM-like pattern and the strong westerly winds [7], consistent with the unprecedented springtime retreat of Antarctic sea ice documented in recent years [15,16]. On the other hand, [12], the regional satellite data described a tendency towards a general decrease in sea ice cover in percentage, probably accompanied by a gradual thinning of ice. This does not coincide with our results from *in situ* observations during the winter of 2011, when the thickness of the sea ice was greater than in the years 2009 (~0.4 m) and 2010 (superficial; overwintering scientists, personal communication).

### Zooplankton succession: Environmental effects vs predator–prey size relationships

The spatial-temporal distribution of zooplankton in PC was first modulated by the dynamics of the environmental variables and then, by trophic relationships. This is evident in the results of the analysis of ordering (cluster), since the composition of zooplankton change in relation of the degree of glacial melting and sea-ice freezing. Additionally, the low values of mesozooplankton in some sampling dates of summer 2011 could be explained by the high levels of turbidity, produced by glacial meltwater. In Antarctic coastal zones, turbidity affects mesozooplanktonic organisms, such as euphausiids and copepods, modifying their distribution, decreasing their ingestion rates and feeding capacity [28,58,59] and producing episodes of mortality [26]. On the other hand, several studies in the WAP showed that food supply is among the most important factors controlling the dynamics of microzooplankton assemblages [18,27,60]. This would indicate that, in our study, the effects of environmental variables on micro- and mesozooplankton biomass were indirect and direct, respectively.

The analysis of the zooplankton succession according to predator-prey size structure allowed identifying trophic relationship scenarios during the different periods, characterized based on the changing environmental conditions. According to Fuentes [47], the impact of the herbivorous food web in PC will depend on which group represents the main primary consumer: copepods or krill/salps, the latter having the greatest impact on phytoplankton. In early summer of 2011, at the beginning of the period of glacial melting, our results showed a possible top-down control on the largest phytoplankton cells in the same sampling dates in which the presence of adult krill or salps was observed. According to Boyd et al. [61], *E*. *superba* is mainly herbivorous during summer in the Bransfield Strait. *S*. *thompsoni* is recorded as the major herbivorous zooplankton in the Southern Ocean [62]. In addition, in summer, the presence of copepods of recognized omnivory could be responsible for the low microzooplankton biomass in the cove. Atkinson [63] showed that the most abundant copepod in our study, *O*. *similis*, ingests small and motile cells almost exclusively before the bloom; while *C*. *propinquus* and *M*. *gerlachei*, consume large cells rather than smaller ones, and showed a preference for motile prey compared with similar sized diatoms. This top-down control by copepods on microzooplankton groups has been previously documented in the Southern Ocean [22,60,64].

Water column stratification is essential for phytoplankton development [12] and dominance of nanophytoplankton cells in Antarctic waters [13,14,65]. Shifts to small phytoplankton cells could also affect the distribution and community composition of microzooplankton [23]. In our study, the observed microzooplankton succession from the smallest to the largest size class would be the result of predator-prey interactions (i.e. possible bottom-up control) observed as a coupling during the period of stratification of the water column. Small athecate dinoflagellates and oligotrichs ciliates graze mainly on the nano- and picoplanktonic size classes [66,67]. During the end of this stratification period the increase in microzooplankton biomass was due to a greater number of tintinnids and a large relative increase of dinoflagellates and copepod nauplii, that have been found to feed on large chain-forming diatoms, other flagellates, and ciliates [22,68,69]. According to Schmoker et al. [21] microzooplankton organisms have similar generation times as phytoplankton, therefore, within short time frames, microzooplankton should catch up with the fast-growing phytoplankton community. Although in our study this association was only statistically observed among the smaller size classes, a coupling of this type has been observed by other authors in Antarctic waters [22].

The succession observed in zooplankton between summer and fall is determined by the change in the role as predator or prey of microzooplankton, which defines the transition from a herbivorous to a microbial food web. The increase in small protozooplankton and large phytoplankton could feed both small and large copepods, as observed in early fall (as a bottom-up control). These circumstances would contribute to the increase of microzooplankton biomass in April due to the lower grazing pressure of copepods on larger microzooplankton. In the Antarctic fall, heterotrophic protozoan biomass would then match or exceed phytoplankton biomass [70]. As previously described, microzooplankton can maintain the phytoplankton biomass at low values, although it can hardly consume the complete bloom [71], so mesozooplankton would also be predating considerably on phytoplankton causing a decrease of its biomass. This could be the situation observed in the fall period in PC, due to consumption by mesozooplankton 10^3^−10^4^ μm on Chl-a <20 and Chl-a >20. At the end of this period, during May, the notable decrease in the biomass of both micro and mesozooplankton is possibly related to the decrease in phytoplankton, added to the selective predation of *C*. *propinquus* on large microzooplankton organisms as documented by Atkinson [63].

The only two previous studies documenting the annual variation of mesozooplankton groups in PC [47,72] registered the highest mesozooplankton abundance when the cove remained frozen. Several studies have registered that after the phytoplankton bloom, different species of copepods such as *O*. *similis*, *C*. *citer* and *C*. *propinquus*, begin to increase their abundance, possibly taking advantage of the availability of different microzooplanktonic groups as potential food resources [63,73,74]. On the other hand, krill furcilia larvae feed within the sea ice but mainly on the primary consumer-grazers of the ice algal community rather than on plankton organisms [75]. Also, during heavy freezing years, the thickness of the sea-ice provides structures that offer protection from predators and food availability for larval krill overwintering [19]. Considering the above, we assume that in PC the high abundance of copepods and furcilia larvae under the sea ice would be due to the presence of available heterotrophic food. It is important to highlight the need for studies of the biota existing within the sea ice and grazing experiments in this period to understand the pelagic food web under the frozen sea at PC.

During the spring succession, the instantaneous response of the microzooplankton to the increase in temperature and availability of food is documented in several studies as an increase in its biomass [76,77]. In our work, the increase in microzooplankton abundance in spring corresponded to small and medium species, which possibly were part of the biota of sea ice. For this reason in PC there was no noticeable increase in microzooplankton biomass during the sea-ice melting. Several authors have documented the important abundance of ciliates and dinoflagellates in the sea ice that when it melting causes an important contribution of these microorganisms to the plankton [70,78,79]. Once again, it could be that the succession of mesozooplankton is due to different feeding strategies of taxonomic groups. First, omnivorous groups with vertical migration capacity mark the increase in mesozooplankton biomass in November. This migration capacity synchronized with the sinking of microalgae provided by the sea-ice melting has been documented for both euphausiids [75] and some copepods [80]. In December, the decrease in mesozooplankton biomass can be attributed to the increase in the representation of predatory carnivores (such as amphipods and hydromedusae) that usually consume copepods [81,82] and salps that are recognized by their herbivory and competition with krill [83].

## Conclusions

During an annual cycle at Potter Cove, mesozooplankton oscillations in abundance and biomass responded to glacier melting (decrease values) and sea-ice freezing (increase values). Under W/NW wind conditions, stratification of the water column benefitted tintinnids development, through an increase in the availability of adequate food-size. The end of the stratification period of the water column is marked by the change in the trophic role of the microzooplankton, from predator to prey defining the transition to a marked microbial food web. Microzooplankton size structure (small species during early summer and larger species during the end of stratification period and early fall) and feeding strategy of mesozooplankton (herbivores during early summer and omnivores during stratification and sea-ice freezing periods) were the main traits which determined the intensity of bottom-up/top-down controls during the temporal succession. If glacier retreat continues and the duration and thickness of the sea ice layer fluctuates as predicted by climate models, our results could be a prediction of future scenarios regarding the zooplankton succession in Antarctic coastal environments.

## Supporting information

S1 Data (XLSX)
